# Usage of social media and Covid 19 vaccine hesitancy among medical students in Kericho County

**DOI:** 10.1371/journal.pgph.0003529

**Published:** 2024-08-22

**Authors:** Calvince Otieno Anino, Philip Sanga

**Affiliations:** 1 Public Health Department, University of Kabianga, Kericho, Kenya; 2 Department of Community Health, Maseno University, Kisumu, Kenya; University of Ghana School of Public Health, GHANA

## Abstract

The recent Covid 19 pandemic revealed the global challenge posed by infectious diseases. Vaccines are a crucial tool in preventing pandemics, as evidenced by their success in controlling past outbreaks. The rise of information and communication technology has introduced social media platforms as potential game-changers in both preventing and managing future pandemics. However, these same platforms can also be a double-edged sword, hindering the uptake of essential vaccination services. This study investigated the association between social media use and vaccine hesitancy among medical students. The study was designed as an institution based cross sectional study of 423 medical students in Kenya Medical Training College and University of Kabianga in Kericho County. Stratified sampling was used to decide on the two institutions and systematic random sampling was used to select the study participants. Research administered questionnaires were used to collect data on the socio-demographic characteristics, use of social media platforms, social media campaigns, and uptake of Covid 19 vaccines. The collected data was analyzed using Statistical Package for Social Sciences version 25. Chi square was used to establish the association between the independent variables and uptake of Covid 19 vaccines. The variables that were significantly associated with hesitancy to Covid 19 vaccines were further analyzed using binary logistic regression. The confidence interval (CI) was set at 95% and statistical significance was considered at *p* < 0.05. The study found significant associations between vaccine hesitancy and several factors, including academic level, preferred social media platform, the influence of social media on attitudes towards vaccines, concerns about vaccine safety and efficacy, and confidence in vaccines. The findings present social media as a potential platform for promotion of vaccines utilization during pandemics when used well.

## Introduction

Hesitancy is defined as the refusal of safe vaccines or delay in acceptance despite its availability [1]. It is a growing global challenge of public health importance, affecting high, middle and low-income countries alike [2]. Surveys conducted by [3, 4] reported that, on average between 40% to 50% of all respondents globally would hesitate to receive Covid-19 vaccine, albeit with wide variations across socio-demographic groups. Hesitancy to Covid 19 vaccine was reported to differ based on age, marital status, gender, level of education and occupation. Higher vaccine hesitancy was reported among people with a lower education level [5, 6], younger age groups [7, 8] and women [7–9]. Additionally, low uptake of Covid 19 vaccine was reported among healthcare workers (HCWs) [10, 11] and medical college students [12, 13]. High hesitancy to Covid 19 vaccines among medical students is an area of concern considering the nature of their training, especially clerkship training which predisposes them to health acquired infections within the hospital settings, and their role as trusted sources of health information [14].

Reasons identified to explain high hesitancy to vaccines were healthcare inequities, unethical research and structural racisms, barriers to access related to marginalization and socioeconomic inequalities such as cost, location and delivery time, and social disadvantages such as lower levels of education and poor access to accurate information [15, 16]. Additionally, inadequate and ineffective public health messages, lack of targeted campaigns, and rumors, conspiracy theories, disinformation, and misinformation through social media were reported to facilitate vaccine hesitancy [1–17]. Investigation into specific factors contributing to Covid 19 vaccine hesitancy reported in literature showed concerns related to convenience, safety, side effects, vaccine efficacy, belief that testing for vaccine was not sufficient, perception that pace of development was too quick, idea that vaccines were not necessary and conviction that vaccine manufacturers were financially motivated [7–18].

According to Merchant and others [19], social media was the primary channel for individuals across various professions to obtain information about health and health interventions. Study [20] among medical students found that Covid 19 vaccine hesitant students were more likely to derive information from social media in contrast to their teachers at the medical college. This is worrisome since social media use was previously associated with misinformation and vaccine hesitancy [21, 22]. Additionally, social media was previously reported to contribute to vaccine hesitancy through conversations, discussions, and campaign based on rumors, disinformation, misinformation and conspiracy theories [23].

The current social media trend among the younger population shows increased use of social media networks and internet to seek information about Covid 19 vaccines [24]. A good proportion of the medical students are within the age range of 18 to 35 years, which coincidentally constitute the most active age category on social media platforms [25]. While unforeseen, there is a potential danger in utilization of health workers who are still in medical school to offer Covid 19 vaccination services. Therefore, it is imperative to investigate the overreliance on social media for information about Covid 19 vaccines among the medical college students and its association with Covid 19 vaccine hesitancy.

## Methods

### Design and setting

The study used institution based cross-sectional design to perform a survey among 423 medical students at Kenya Medical Training College (KMTC) and University of Kabianga. Kericho County is one of the 47 counties in Kenya. It is a host to five medical colleges and universities, that is Sigowet KMTC, AIC Litein Medical Training College, Kenya Highland Evangelical University, KMTC Kapkatet Campus and University of Kabianga. Simple random sampling was used to decide on KMTC Campus and University of Kabianga. Further, stratified sampling was used to select the study participants based on their academic levels, 1^st^ year, 2^nd^ year and 3^rd^ year. Thereafter, students were randomly selected based on their class list. Probability proportionate to size was used to allocate students from both institutions. Research administered questionnaires were used to collect data on socio-demographic characteristics, use of social media platforms for conversations and discussions, social media campaigns, and hesitancy to Covid 19 vaccines. Data collection was carried out from May 1^st^, 2023 to September 30^th^, 2023.

### Variables

#### Outcome variable

The study used a single item measure with four possible responses as described by [26] to measure hesitancy to Covid 19 vaccine. Accepting the vaccine without doubt and accepting the vaccine with doubt responses were further computed to ‘No’ response. Refusing the vaccine or intention to delaying the vaccine was computed to ‘Yes’ response. Hesitancy was therefore measured with either a ‘Yes’ or ‘No’ response.

#### Independent variables

Social media platform usage was assessed across two categories. The first category focused on the utilization of social media as a conversational platform for discussing vaccination services. The second classification centered on the use of social media as campaign platforms specifically related to Covid-19 vaccination. The themes considered to have influence on Covid 19 vaccine hesitancy that were discussed and campaigned for or against were grouped based on the 3Cs antecedent model on vaccination [26]. The participants profile assessed in the study are presented in Table 1.

**Table 1:** Participants profile on social media usage and discussed themes.

Category	Description
Socio-demographics	Age, gender, and academic level at medical school
Vaccine hesitancy	Categorical rating of the participant’s hesitancy towards vaccines
Conversational platform	Platforms used, frequency of use, preferred platform, and perception on influence of platform used on attitude
Campaign platform	Platform used, influence of campaigns on perception about vaccines, and extent campaigns influenced attitude
Themes discussed and/or campaigned for/against	**Confidence:** Safety of the vaccine, efficacy of the vaccine, government recommendations, advise from healthcare professionals, religious or cultural beliefs, and political beliefs **Complacency:** Influence of family and friends, lack of information about the vaccine **Convenience:** Availability of the vaccine, and lack of access to the vaccine

#### Data analysis

The collected data was analyzed using Statistical Package for Social Sciences version 25. Age variable was transformed from a continuous variable to a categorical variable. The categorization was determined by whether the participant fell into the youth category, which was defined as individuals between 18 to 35 years, or above 35 years. Chi square was thereafter used to establish the association between the independent variables and uptake of Covid 19 vaccines. Additionally, themes that were campaigned for or against that were statistically associated with confidence were further consolidated to a single variable which represented confidence in vaccines. The variables that were significantly associated with uptake of Covid 19 vaccines were further analyzed using binary logistic regression. They included, academic level, use of social media platform to discuss about Covid 19 vaccine, participation in social media campaigns related to Covid 19 vaccine, thought that social media influenced one’s attitude towards Covid 19 vaccine and the extent of influence. Concerns about vaccine safety, concerns about vaccine efficacy and lack of information about the vaccines were the themes discussed in social media platforms that were moved to binary logistic regression. Confidence in vaccines and influence of family and friends were also considered for regression. The results were reported as adjusted odds ratio (AoR). The confidence interval (CI) was set at 95% and statistical significance was considered at *p* < 0.05.

#### Ethical consideration

The study was approved by the Institutional Scientific Review Committee of the University of Kabianga and was assigned approval number IERC/2023/008. All the participants were individually informed about the research including the purpose of carrying out the research, the potential benefits of participating in the study, and the assurance that no harm would arise from their participation. Additionally, both verbal and written consent was sought from the respondents to ensure that their participation was voluntary. Privacy and confidentiality were also ensured in the study. Privacy was ensured by interviewing the participants in a designated private room within the participating institution. The private rooms provided a confidential space for open communication. With regards to confidentiality, the data was collected through the use of Kobo Collect. All the information obtained was securely submitted to a central server hosted in the Kobo Collect. Access to this server was restricted to the research team members exclusively. Moreover, anonymity of the respondents was ensured by assigning them unique codes instead of using their names. This was done for all the study participants and throughout the study. This approach was preferred since it safeguarded the privacy of the participants and created a trustworthy research environment. It indeed ensured that the identity of participants remained confidential.

## Results

### Socio-demographic characteristics

Overall, 35.8% of the respondents displayed hesitancy to Covid 19 vaccines. Among the factors studied that influenced hesitation was the socio-demographic characteristics as shown in Table 2. The study focused on three socio-demographic characteristics which included age, gender and academic level in medical school. The latter was significantly associated with hesitancy (p = 0.02), while age and gender were not. The largest proportion of hesitancy, at 41.1%, was observed among first-year students, followed by third-year students and second year students respectively.

**Table 2:** Socio-demographic characteristics of the respondents.

Variables	Hesitancy		P value
	Yes	No	
	151 (35.8%)	272 (64.2%)	
Age			0.50
18 to 35 years	151 (100)	264 (97.1)	
Above 35 years	-	8 (2.9)	
Gender			0.55
Female	54 (35.8)	124 (45.6)	
Male	97 (64.2)	148 (54.4)	
Academic level at medical school			0.01
1^st^ year	62 (41.1)	78 (28.7)	
2^nd^ year	43 (28.5)	128 (47.1)	
3^rd^ year	46 (30.5)	66 (24.3)	

*P* = < 0.05, 95% confidence interval

### Social media conversation platforms

We observed significant associations between the choice of social media platform used for discussing Covid 19 vaccine and vaccine hesitancy (p = 0.02) as shown in Table 3. Additionally, our study found significant association between social media influence on attitude and vaccine hesitancy (p<0.001). The preference for a specific social media platform was associated with varying levels of hesitancy towards Covid-19 vaccines. For instance, TikTok and Instagram with 64.3% and 62.5% had the highest proportion of respondents expressing hesitancy compared to 35.7% and 37.5% of those who did not. Conversely, Facebook, X (formerly known as twitter) and WhatsApp had the largest proportion of respondents not expressing hesitancy compared to those expressing hesitancy as shown in Table 2. A small proportion (31.6%) of the respondents whose attitude about Covid 19 vaccines was influenced by social media expressed hesitancy compared to those who did not (44.2%). Use of social media and frequency of using social media for discussion of Covid 19 vaccines were not statistically associated with vaccine hesitancy.

**Table 3:** Association between usage of social media as discussion platforms on vaccination services and Covid 19 vaccine hesitancy.

	Hesitancy		P value
	Yes	No	
Use social media to discuss Covid 19 vaccine			0.10
Yes	116 (33.2)	233 (66.8)	
No	35 (47.3)	39 (52.7)	
Frequency of using social media platform to discuss Covid 19 vaccine			0.48
Always	20 (26.3)	56 (73.7)	
Sometimes	83 (35.9)	148 (64.1)	
Never	13 (31)	29 (69)	
Priority social media platform used to discuss Covid 19 vaccines			0.02
Facebook	27 (21.4)	99 (78.6)	
X (formerly known as twitter)	45 (37.8)	74 (62.2)	
Instagram	10 (62.5)	6 (37.5)	
WhatsApp	25 (33.8)	49 (66.2)	
Tik tok	9 (64.3)	5 (35.7)	
Social media platform influenced attitude about Covid 19 vaccines			< 0.001
Yes	97 (31.6)	209 (68.4)	
No	19 (44.2)	24 (55.8)	

*P* = < 0.05, 95% confidence interval

### Social media campaign platforms

Participation in social media campaigns (<0.001) and the extent to which social media was used as a platform for campaign (0.001) were statistically associated with vaccine hesitancy as shown in Table 4. A small proportion (25.0%) of those who used social media as a campaign platform for Covid 19 vaccines were hesitant to receive vaccines. In contrast, 53.5% among those who did not participate in social media campaigns were hesitant to receive Covid 19 vaccines. Extent social media campaigns influenced attitude varied across the respondents. The resulting effect on hesitancy differed with the extent of variation on attitude. Respondents who reported a great deal influence had the highest hesitancy (34.1%), followed by a little (30.6%) and somewhat influence (20.7%). However, there was no hesitancy among those whose attitude was not influenced by social media campaigns.

**Table 4:** Association between usage of social media as campaign platforms on vaccination services and Covid 19 vaccine hesitancy.

	Hesitancy		P value
	Yes	No	
Participated in social media campaigns related to Covid 19 vaccine			< 0.001
Yes	66 (25.0)	198 (75.0)	
No	85 (53.5)	74 (46.5)	
Priority social media platform for campaigns on Covid 19 vaccine			0.18
Facebook	23 (27.1)	62 (72.9)	
X (formerly known as twitter)	39 (31.6)	85 (68.5)	
WhatsApp	4 (9.3)	39 (90.7)	
Tik tok	-	12 (100)	
Influence of social media campaigns on perception about Covid 19 vaccines			0.44
Increased knowledge about Covid 19 vaccines	48 (26.4)	134 (73.6)	
Changed attitude about Covid 19 vaccines	14 (28.6)	35 (71.4)	
Encouraged me to get vaccinated	-	21 (100)	
Did not impact my perspective on Covid 19 vaccines	4 (33.3)	8 (66.7)	
Extent social media campaigns influenced attitude about Covid 19 vaccines			0.001
A great deal	28 (34.1)	54 (65.9)	
Somewhat	23 (20.7)	88 (79.3)	
A little	15 (30.6)	34 (69.4)	
Not at all	-	22 (100)	

*P* = < 0.05, 95% confidence interval

### Social media discussion themes

Themes discussed on social media platforms that were statistically associated with vaccines hesitancy included concerns about vaccine safety (p = 0.03), concerns about vaccine efficacy (p = 0.01) and lack of information about the vaccines (0.01) as shown in Table 5. A bigger proportion (51.5%) of the respondents who were concerned about vaccine safety were more likely to express hesitancy to vaccines compared to 22.2% who were not. A similar pattern was observed among those who lacked information about Covid 19 vaccines with 45.1% who didn’t have the information expressing hesitancy to vaccines compared to 33.4% who had the information. Additionally, lightly over a third of the respondents (36%) who were concerned about the efficacy of the vaccines, and nearly a similar number of respondents (35.4%) who were not concerned expressed hesitancy to Covid 19 vaccines. However, variables such as religious or cultural beliefs, political beliefs and lack of access to the vaccines were not statistically associated with vaccine hesitancy.

**Table 5:** Themes that were discussed on the social media platforms.

Variables	Hesitancy		P-value
	Yes	No	
Concerns about vaccine safety			0.03
Yes	101 (51.5)	95 (49.5)	
No	50 (22.0)	177 (78.0)	
Concerns about vaccine efficacy			0.01
Yes	72 (36.0)	128 (64.0)	
No	79 (35.4)	144 (64.6)	
Religious or cultural beliefs			0.33
Yes	48 (26.7)	132 (73.3)	
No	103 (42.4)	140 (57.6)	
Political beliefs			0.26
Yes	39 (36.4)	68 (63.6)	
No	112 (35.6)	204 (64.4)	
Lack of information about the vaccine			0.01
Yes	37 (45.1)	45 (54.9)	
No	114 (33.4)	227 (66.6)	
Lack of access to the vaccine			0.14
Yes	27 (36.5)	47 (63.5)	
No	124 (35.5	225 (64.5)	

*P* = < 0.05, 95% confidence interval

### Social media campaign themes

We evaluated confidence in the vaccine through an analysis of the safety of the vaccine, its efficacy, government recommendations, and advice from health professionals, political beliefs and religious or cultural beliefs as presented in Table 6. These themes were assessed within the framework of the 3Cs antecedents to vaccination described by [26]. Other than political beliefs and religious or cultural beliefs, the results indicated a statistically significant association between all the themes studied and vaccine hesitancy. The statistically significant variables were consolidated to a new variable ‘confidence in vaccine’. The variable on confidence in vaccine was identified to be significantly associated with vaccine hesitancy (p = 0.03). Among the respondents who displayed hesitancy, 62% had no confidence in the vaccine compared to 19.9% who expressed hesitancy despite having confidence in vaccines. Additionally, influence of family and friends was also found to be statistically associated with vaccine hesitancy (p = 0.01). A significant majority (59.1%) of the respondents who expressed hesitancy were influenced by families and friends, whereas only a small proportion (31.1%) who were not influenced by families and friends displayed hesitancy.

**Table 6:** Themes that were campaigned for or against on the social media platforms.

	Hesitancy		P-value
	Yes	No	
Safety of the vaccine			< 0.001
Yes	70 (22.8)	239 (77.2)	
No	81 (71.1)	33 (28.9)	
Efficacy of the vaccine			0.04
Yes	43 (32.1)	91 (67.9)	
No	108 (37.4)	181 (62.6)	
Government recommendations			0.02
Yes	65 (28.8)	161 (71.2)	
No	86 (43.7)	111 (56.3)	
Advice from healthcare professionals			0.01
Yes	33 (12.4)	234 (87.6)	
No	118 (75.6)	38 (24.4)	
Religious or cultural beliefs			0.17
Yes	86 (48.6)	91 (51.4)	
No	65 (26.4)	181 (73.6)	
Political beliefs			0.22
Yes	128 (45.7)	152 (54.3)	
No	23 (16.1)	120 (83.9)	
Confidence in vaccine			0.03
Yes	53 (19.9)	212 (80.1)	
No	98 (62.0)	60 (38.0)	
Influence of family and friends			0.01
Yes	41 (59.1)	28 (40.9)	
No	110 (31.1)	244 (68.9)	
Availability of the vaccine			0.35
Yes	107 (53.6)	92 (46.4)	
No	44 (19.6)	180 (80.4)	

*P* = < 0.05, 95% confidence interval

### Social media influence and vaccine hesitancy

The study observed a likelihood of vaccine hesitancy based on several factors, including academic level, priority social media platform, influence of social media on attitude, concerns about vaccine safety and efficacy, and confidence in vaccines as shown in Table 7. Students in their second year of study were twice as likely to express hesitancy to vaccines than their counterparts in year three. We also found that Facebook and WhatsApp users were less likely to express hesitancy to vaccines compared to X (formerly known as twitter). The study further observed a fourfold likelihood of hesitancy among individuals whose attitude was influenced by themes discussed and campaigned for or against in the social media platforms. Additionally, higher odds of hesitancy to vaccines were observed among social media users who had a discussion on concerns about vaccine safety and efficacy. However, those who held campaigns to bolster confidence in vaccines were less likely to express hesitancy to vaccines compared to those who did not.

**Table 7:** Binary logistic regression analysis of social media related factors and Covid 19 vaccines hesitancy.

Variable	*P*	AoR	95% CI
Academic level			
1^st^ year	0.78	0.9	0.52–1.22
2^nd^ year	0.04	2.1	1.64–2.78
3^rd^ year	1		
Priority social media platform used to discuss Covid 19 vaccines			
X (formerly known as twitter)	1		
Facebook	0.04	0.8	0.71–1.16
Instagram	0.10	3.1	2.65–4.93
WhatsApp	0.02	0.6	0.49–7.88
Tik tok	0.12	1.6	1.26–2.04
Social media has influenced attitude towards Covid 19 vaccine			
No	1		
Yes	0.01	4.6	3.03–5.27
Participated in social media campaigns related to Covid 19 vaccine			
No	1		
Yes	0.17	3.0	2.50–3.74
Extent social media campaigns have influenced attitude			
A great deal	1		
A little	0.54	0.8	0.71–0.99
Not at all	0.79	1.1	0.93–1.26
Somewhat	0.99	4	2.66–5.98
Concern about vaccine safety			
No	1		
Yes	0.02	2.2	1.75–3.41
Concern about vaccine efficacy			
No	1		
Yes	0.01	1.5	1.23–1.68
Lack of information about vaccine			
No	1		
Yes	0.89	0.7	0.48–1.32
Confidence in vaccine			
No	1		
Yes	0.01	0.4	0.11–0.78
Influence of family and friends			
No	1		
Yes	0.42	0.9	0.73–1.25

1 –reference category, AoR–adjusted odds ratio, CI–confidence interval, model adjusted for academic level at medical school

## Discussion

Our findings on vaccine hesitancy (35.8%) are consistent with a previous study among healthcare workers in our region, which reported a similar hesitancy level of 37% [7]. However, research from other areas showed significant variation in hesitancy among student populations. One study found low hesitancy (13%) among students [27], while others reported higher hesitancy levels among medical students in different regions (46% and 20% in one study, 45% in another) [28, 29]. Like the general population, vaccine utilization and hesitancy levels vary greatly among healthcare students based on their location, heterogeneity and their training courses [30]. Our study further observed significant variations in vaccine hesitancy levels based on academic level of the students. High hesitancy levels to Covid 19 vaccines of about 47% was reported among senior medical students [28]. Additionally, high intention to hesitancy to vaccines was observed among senior medical students in the United States [31]. Our findings were contrary to the observed trend, as the highest hesitancy was identified among first-year students in our study. Additionally, while fewer second-year students were noted to exhibit hesitancy towards vaccines, they displayed double the odds of hesitancy compared to the senior students in year three. The difference in hesitancy across the academic levels could be attributed to different course contents in the students’ curricula or from the observation that similar courses are handled at different levels from one country to another [32, 33]. This observation is of great significance since senior medical students are prepared to help medical teams and often are allowed to practice as apprentice medical workers [34]. They should therefore adhere to all Covid 19 guidelines including protection through PPEs and vaccination since these are crucial steps to preventing health acquired infections [35].

Attitudes towards Covid-19 vaccines was greatly influenced by social media platforms. When we compare our findings with earlier works, we found varied patterns. Our findings were similar to those reported by [36, 37] who explored the impact of social media discussions on vaccine perceptions. Our concurrence was on the premise that information shared on social media may affect public attitude towards Covid 19 vaccines. However, we disagreed with [37] who didn’t find association between social media usage factors and hesitancy towards vaccines. Our study observed that priority social media platform was a significant player which shaped individuals’ hesitancy towards vaccines. We reported lower odds of hesitancy among individuals who preferred WhatsApp and Facebook as opposed to those who preferred using X (formerly known as twitter) to discuss Covid 19 issues. Our finding disagreed with [38] who reported low Measles Rubella vaccination acceptance among parents who had trust in social media and WhatsApp information. The nature of information dissemination among WhatsApp and Facebook users in our study was the basis of our argument for the lower odds of hesitancy among medical students. In both platforms users can discuss issues in closed groups which are more private and widely accepted for personal communications. Additionally, closed groups enable information sharing among trusted connections only [39] and are therefore mostly used among family and friends. Close knit social network was reported [40] as a contributing factor to a positive influence on vaccine acceptance.

However, our finding raises intriguing questions about the role of different social media platforms in shaping vaccine attitudes. Earlier studies reported X as the most widely used platform for conversations and presenting public opinion about Covid 19 vaccination [41, 42]. Indeed, a surge among a diverse range of users of X such as opinion leaders and influencers were observed at the beginning and peak of the pandemic [18]. The preference for X among these users may be attributed to the open and fast-paced nature of information exchange on X [43]. Furthermore, X is at the forefront in dissemination of information and provision of real-time updates which is desired by users [44]. However, these functions are beyond WhatsApp and Facebook platforms. Therefore, conversations within private spaces are potentially the reasons for the reduced hesitancy among the WhatsApp and Facebook users since they foster a sense of trust and credibility. Our findings therefore identify WhatsApp and Facebook as possible platforms to counter misinformation and enhance positive vaccine perceptions and acceptance.

Among the predictors of vaccine hesitancy described by [26] confidence in vaccine was the sole dimension that was statistically associated with both low and high hesitancy. The specific indicators of confidence we identified for the increased likelihood of vaccine hesitancy were concerns about vaccine safety and concerns about efficacy. Our findings concurred with earlier reports by [28] who reported that 56% of respondents had expressed fear of adverse effects from Covid-19 vaccines. In the same study, 40% and 23% of the respondents had doubts regarding Covid 19 vaccine safety and effectiveness, respectively.

Interestingly, we also observed that confidence in vaccines was a facilitator for increased utilization of Covid 19 vaccine as it was associated with low odds for hesitancy. The specific indicators of confidence in vaccines which could have shaped the interactions and experiences of medical college students within online platforms were both safety and efficacy to vaccines, government recommendations and advice from healthcare professionals. Our findings aligned with previous reports by [45] which showed high acceptance of Covid 19 vaccines among individuals who had trust in experts and leaders, among those who had high perception on government measures and recommendations [6], and among those who had trust in the efficacy of the vaccine [8]. Additionally, the confidence expressed by individuals in the study was probably influenced by positive experiences with healthcare providers and their recommendations. Our stance is grounded in the frequent interactions that medical college students have with healthcare workers during their clinical rotations and apprenticeship. These engagements provide ample opportunities for positive influences on their perspectives and attitudes.

Therefore, one of the key strengths of the current study is that it explored the impact of different social media platforms on vaccine attitudes. This is important as social media plays a major role in shaping public opinion. Additionally, the finding that closed groups on WhatsApp and Facebook might be associated with lower hesitancy is important since it’s an indicator that these platforms can be targeted for information campaigns. However, our study was delimited to two medical colleges in Kenya. This implies that generalization of the findings should be carried out with caution since the study sample may not fully represent the diversity of medical students across various regions. Nevertheless, our choice of the study institutions was such that the two institutions studied represented the two major strata of medical institutions in Kenya, which are medical training college and university. This enhanced representativeness of our sample. Furthermore, the study primarily focused on online platforms of social media as a factor influencing Covid 19 vaccine hesitancy. This may have limited exploration to traditional media and interpersonal communications. To counter this weakness, we developed a study tool that had extensive range of influence on vaccine attitudes.

## Conclusion

In conclusion, this study found high vaccine hesitancy level among medical students, with variations depending on their year of study. Interestingly, though social media is known to disinform and misinform, students who used Facebook and WhatsApp were less hesitant, which is an indication that these platforms could be valuable tools for promoting vaccination. Finally, the study observed the potential influence of social media in building confidence in vaccine safety, efficacy, and endorsements from trusted sources like governments and healthcare professionals. By focusing on these elements in targeted social media communication, we can improve vaccine acceptance among future healthcare workers.

### Recommendation

Public health agencies, medical professional associations, and educational institutions can reduce vaccine hesitancy by using the influence of social media platforms like Facebook and WhatsApp through targeted campaigns on vaccine safety, efficacy, and endorsements from trusted sources like government health bodies and healthcare professionals.

## Supporting information

S1 Data Data on the study variables. (CSV)
